# Super-Selective Embolization Using Flow-Directed Microcatheter and 0.010-inch Microwire for Type II Endoleak Following Thoracic Endovascular Aortic Repair: A Three-Case Report

**DOI:** 10.1016/j.radcr.2025.09.040

**Published:** 2025-10-04

**Authors:** Hiroki Kamada, Sota Oguro, Hiromitsu Tannai, Hiroyuki Sakakibara, Kei Takase

**Affiliations:** Department of Diagnostic Radiology, Tohoku University Hospital, Sendai, Japan

**Keywords:** Type II endoleak, Thoracic endovascular aortic repair, Endovascular embolization, Flow-directed microcatheters, N-butyl cyanoacrylate

## Abstract

Type II endoleak (T2EL) following thoracic endovascular aortic repair (TEVAR) may lead to progressive aneurysm enlargement, necessitating further intervention. This report presents 3 cases of persistent T2EL with aneurysm growth after TEVAR, and managed through endovascular embolization using flow-directed microcatheters and 0.010-inch guidewires, employing N-butyl cyanoacrylate (NBCA) either alone or in combination with coils. Aneurysmal sac embolization was successfully performed in all cases, despite significant vascular tortuosity, resulting in favorable immediate outcomes without complications. A key advantage observed was the ability of flow-directed microcatheters to navigate into distal, tortuous feeding vessels that are typically inaccessible using conventional methods. While NBCA alone was effective for embolizing small sacs, a combination of coils and NBCA provided more controlled and stable embolization in larger sacs. However, follow-up computed tomography revealed new endoleaks in 2 cases, emphasizing the need for continued monitoring. While short-term results are promising, further studies are needed to assess long-term recurrence risks. This case series highlights the effectiveness of flow-directed microcatheters, 0.010-inch microwires, and targeted embolization techniques in managing T2EL following TEVAR.

## Introduction

Thoracic endovascular aortic repair (TEVAR) has seen rapid adoption due to its minimally invasive nature and expanding therapeutic indications; however, post-operative endoleaks remain a significant clinical challenge. Although type II endoleak (T2EL) occurs relatively infrequently following TEVAR [1,2], persistent cases can lead to aneurysm enlargement, requiring further interventions. The primary mechanism of T2EL following TEVAR is retrograde blood flow from small-caliber and tortuous branch vessels, such as the subclavian artery branches, intercostal arteries, and bronchial arteries, which complicates endovascular management [[3], [4], [5], [6]].

Available treatments include endovascular embolization, direct puncture, and surgical repair. Direct puncture carries risks such as pneumothorax and bleeding due to the traversal of thoracic structures [7]. Surgical repair is more invasive and associated with higher perioperative risks. Endovascular embolization, on the other hand, is often complicated by the difficulty of navigating catheters through small, tortuous vessels. Embolization strategies include feeder-only embolization and embolization of the aneurysm sac. Some studies indicate that sac embolization offers superior aneurysm stabilization and endoleak resolution [8], while others report no significant difference compared to feeder-only embolization [9]; thus, a clear consensus has not yet been established. It should also be noted that branches of the subclavian and bronchial arteries may communicate with spinal arteries. Therefore, proximal embolization limited to the feeder vessels must be approached with caution due to the risk of spinal ischemia.

In this report, we present 3 cases of embolization of T2EL associated with aneurysm enlargement occurring during long-term follow-up after TEVAR. Endovascular embolization was successfully performed using small-caliber, highly flexible, flow-directed microcatheters guided by 0.010-inch microwires. The embolic material used was N-butyl cyanoacrylate (NBCA), either alone or in combination with coils. Despite technical challenges, favorable short-term outcomes were achieved in all cases.

## Case presentation

This retrospective case series includes 3 patients who underwent embolization for T2EL following TEVAR at our institution. The study was approved by the ethics committee, and informed consent was obtained from all patients, both verbally and in writing.

### Case 1

**Fig. 1:**
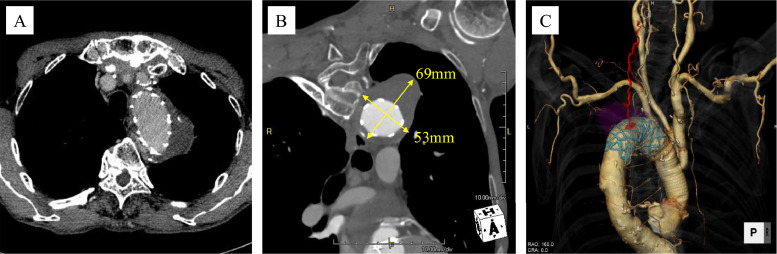
Computed tomography (CT) in Case 1. (A) A type II Endoleak (T2EL) demonstrated in the proximal descending aorta. (B) Maximum diameter of the aneurysm enlarged to 69 mm. (C) Feeding vessels originated from the left intercostal artery, deep cervical artery, costocervical artery, and left subclavian artery.

A 69-year-old man had previously undergone ascending and arch aortic replacement with open stent graft insertion and bioprosthetic aortic valve replacement at another institution, performed 1 year earlier for chronic type B aortic dissection. Post-operative computed tomography (CT) at 6 months demonstrated a persistent T2EL with a 4 mm increase in aneurysm size, reaching a diameter of 60mm. Subsequent CT imaging at our hospital revealed continued endoleak in the proximal descending aorta, with further aneurysm enlargement to a maximum diameter of 69 mm (Fig. 1A and B). The feeding vessels were identified as arising from the left intercostal, deep cervical, costocervical, and left subclavian arteries (Fig. 1C). Based on the progressive aneurysm enlargement and imaging findings; endovascular embolization was indicated.

**Fig. 2:**
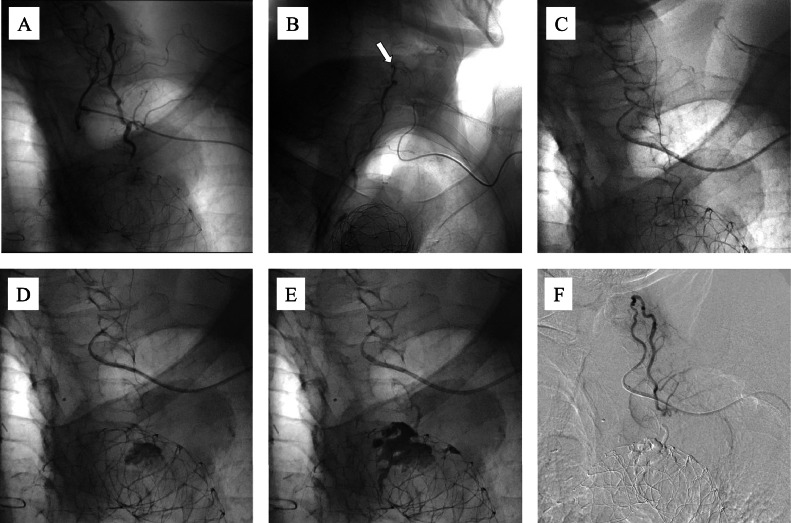
Embolization procedure in Case 1. Endovascular embolization of a type II Endoleak (T2EL) in case 1. A 4-Fr sheath was inserted into the left brachial artery. (A) Digital subtraction angiography (DSA) identifying the feeder vessel as the aneurysmal sac arising from the costocervical artery, deep cervical artery, and responsible feeder vessel. (B) A triple coaxial system comprising 2.85/2.9-Fr (Carry Leon High-flow, UTM, Aichi, Japan) and 1.5/1.7/1.9-Fr (Carry Leon 2 marker, UTM, Aichi, Japan) microcatheters, and a 0.014-inch (Meister, Asahi Intecc, Aichi, Japan) guidewire was inserted and advanced toward the aneurysm. However, the 1.5/1.9-Fr microcatheter could not be advanced distally beyond the proximal segment of the deep cervical artery (white arrow). (C) Microcatheter system was changed to a 6.5-Fr guiding sheath. Using a VTA catheter and a 0.010-inch (CHIKAI 10, Asahi Intecc, Aichi, Japan) guidewire, a 1.5/2.4-Fr (DeFrictor BULL, Medicos Hirata, Tokyo, Japan) flow-directed microcatheter was inserted, successfully advancing from the deep cervical artery into the intercostal artery and finally reaching the aneurysm sac. (D, E) DSA demonstrated an aneurysm sac measuring approximately 18 mm in diameter. Embolization was performed using approximately 1.5 mL NBCA mixed with lipiodol (33%). (F) Post-embolization DSA confirmed elimination of the endoleak.

For the endovascular procedure, the left brachial artery was punctured, and a 4-Fr sheath was introduced. A 4-Fr VTA catheter was advanced into the subclavian artery using a 0.035-inch guidewire. Digital subtraction angiography (DSA) identified the feeder vessels to the aneurysmal sac as the costocervical artery, deep cervical artery, and the primary responsible feeder (Fig. 2A). A triple coaxial system comprising 2.85/2.9-Fr (Carry Leon High-flow, UTM, Aichi, Japan) and 1.5/1.7/1.9-Fr (Carry Leon 2 marker, UTM, Aichi, Japan) microcatheters, and a 0.014-inch (Meister, Asahi Intecc, Aichi, Japan) guidewire was inserted and advanced toward the aneurysm. However, the 1.5/1.9-Fr microcatheter could not be advanced distally from the proximal portion of the deep cervical artery (Fig. 2B). Due to the difficulty in accessing the aneurysm sac, the microcatheter system was replaced with a 6.5-Fr guiding sheath. Using a VTA catheter and a 0.010-inch (CHIKAI 10, Asahi Intecc, Aichi, Japan) guidewire, a 1.5/2.4-Fr (DeFrictor BULL, Medicos Hirata, Tokyo, Japan) flow-directed microcatheter was successfully advanced from the deep cervical artery into the intercostal artery and ultimately into the aneurysm sac (Fig. 2C). DSA revealed an aneurysm sac, measuring approximately 18 mm in diameter. Embolization was performed using approximately 1.5 mL of NBCA mixed with lipiodol (33%) (Fig. 2D and E). Subsequent DSA confirmed elimination of the endoleak (Fig. 2F).

A follow-up CT scan at 1 month confirmed no recurrence of endoleak or aneurysm enlargement.

### Case 2

A 44-year-old woman with systemic lupus erythematosus (SLE) underwent thoracoabdominal aortic replacement for an abdominal aortic aneurysm 5 years earlier, followed by ascending and aortic arch replacement with subsequent TEVAR for a thoracic aortic aneurysm 4 years ago. Immediately after TEVAR, CT revealed a T2EL originating from the intercostal arteries within the proximal descending aorta, with an associated aneurysm measuring approximately 50 mm in diameter.

**Fig. 3:**
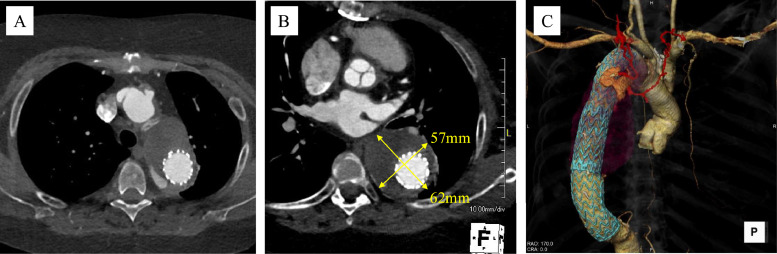
Computed tomography (CT) in Case 2. (A) CT revealed persistent endoleak in the proximal descending aorta. (B) The aneurysm gradually enlarged over a period of 3 years, reaching a maximum diameter of approximately 62 mm. (C) Feeding vessels included bilateral intercostal arteries originating from the thyrocervical arteries.

Follow-up CT revealed a persistent endoleak with gradual aneurysmal enlargement of 7 mm over 3 years, reaching a maximum diameter of approximately 62 mm (Fig. 3A and B). CT angiography indicated that the bilateral intercostal, thyrocervical, and subclavian arteries were involved as feeder vessels (Fig. 3C).

**Fig. 4:**
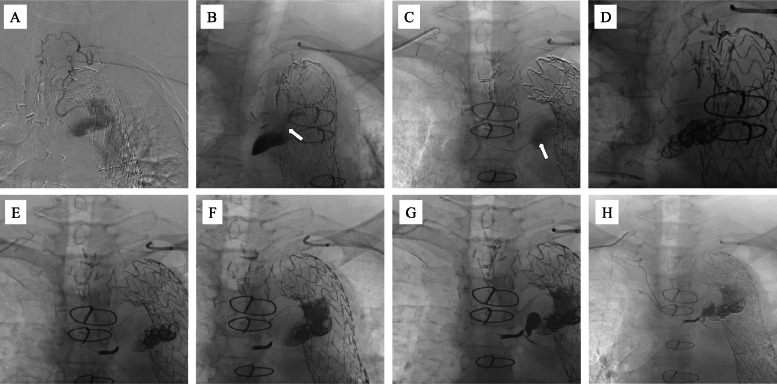
Embolization procedure in Case 2. Both brachial arteries were punctured, with a 5.3-Fr guiding sheath inserted on the left side and a 4-Fr sheath on the right side. (A) Left thyrocervical artery is selected using a 4Fr VTA catheter. Digital subtraction angiography (DSA) revealed an aneurysmal sac via the intercostal artery. (B) A 1.5/2.4-Fr (DeFrictor BULL, Medicos Hirata, Tokyo, Japan) microcatheter was navigated through the tortuous vessels using 0.010-inch (CHIKAI 10 and CHIKAI X010, Asahi Intecc, Aichi, Japan) guidewires, successfully reaching the aneurysm sac (white arrow). (C) Right thyrocervical artery was similarly accessed using a 4-Fr RIM catheter and a 1.5/2.4-Fr (DeFrictor BULL, Medicos Hirata, Tokyo, Japan) microcatheter, which also reached the sac (white arrow). (D) Six 0.012-inch (i-ED, Kaneka Medix. Corporation, Osaka, Japan) coils were placed in the aneurysm sac using the right microcatheter system. (E) Three additional coils were placed in the intercostal artery. (F) On the left side, a mixture of N-butyl cyanoacrylate (NBCA) and lipiodol (33%) was injected to fill the sac. (G) NBCA embolization was performed on the right side following a lidocaine test at the feeder vessel, confirming the absence of neurological symptoms. (H) DSA confirms successful embolization of both the feeding arteries and the aneurysmal sac.

Both brachial arteries were accessed, with a 5.3-Fr guiding sheath inserted on the left side and a 4-Fr sheath on the right. The left thyrocervical artery was catheterized using a 4-Fr VTA catheter. DSA revealed an aneurysmal sac supplied via the intercostal artery (Fig. 4A). A 1.5/2.4-Fr (DeFrictor BULL, Medicos Hirata, Tokyo, Japan) microcatheter was navigated through tortuous vessels using 0.010-inch (CHIKAI 10 and CHIKAI X010, Asahi Intecc, Aichi, Japan) guidewires, successfully reaching the sac (Fig. 4B). The right thyrocervical artery was similarly catheterized using a 4-Fr RIM catheter and a 1.5/2.4-Fr (DeFrictor BULL; Medicos Hirata, Tokyo, Japan) microcatheter, which also reached the aneurysm sac (Fig. 4C).

DSA revealed a large aneurysm sac measuring approximately 50 mm in diameter. As coil delivery was deemed feasible, embolization using a combination of coils and NBCA was planned. Through the right microcatheter system, six 0.012-inch (i-EDs, Kaneka Medix Corporation, Osaka, Japan) coils were deployed within the aneurysm sac (Fig. 4D). Although the Defrictor BULL microcatheter has a narrow inner diameter (0.013–0.015-inch), the coils were successfully advanced. However, due to severe vessel tortuosity, inserting the 0.014-inch coil delivery pusher proved difficult. Therefore, the coils were detached within the microcatheter and manually delivered using a 1 mL syringe filled with water. Subsequently, 3 additional coils were inserted into the intercostal artery feeder (Fig. 4E).

NBCA mixed with Lipiodol (33%) was injected from the left side to fill the aneurysmal sac (Fig. 4F). DSA confirmed complete embolization of both the sac and the feeder vessels. However, residual contrast enhancement was observed through coil gaps on the right-sided catheter. Consequently, NBCA embolization was also performed on the right side after a lidocaine test at the feeder confirmed the absence of neurological symptoms (Fig. 4G). Final DSA confirmed embolization of the feeders and the aneurysm sac (Fig. 4H).

A follow-up CT at 6 months revealed successful embolization. Although a new T2EL from a different intercostal artery was identified, no aneurysm enlargement was observed, and conservative follow-up was continued.

### Case 3

**Fig. 5:**
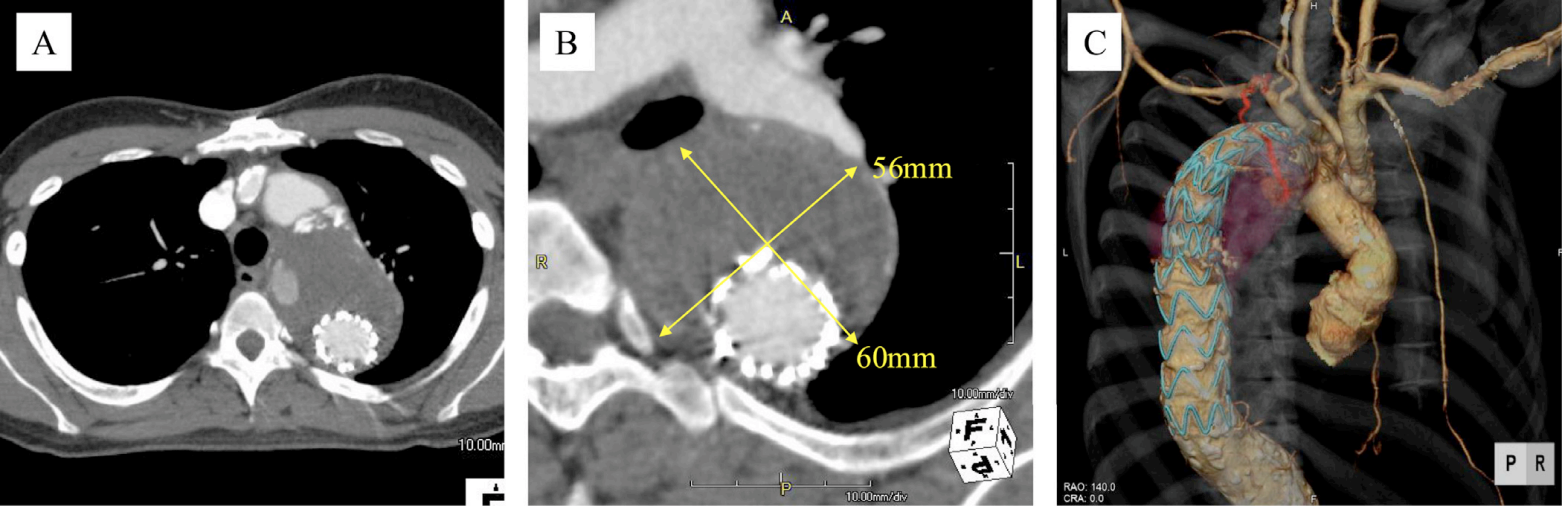
Computed tomography (CT) in Case 3. (A) An endoleak was identified in the proximal ascending aorta. (B) Aneurysm enlarged to approximately 60 mm in diameter (2 mm enlargement over 6 months). (C) Feeder vessel coursed along the left vertebral body and formed connections with the bronchial artery, costocervical artery, and left subclavian artery.

A 34-year-old woman had undergone ascending aortic arch replacement 13 years earlier for an ascending aortic aneurysm secondary to Takayasu arteritis. Due to a 5 mm increase in the diameter of the descending aorta over 3 years, TEVAR was performed 1 year prior. Follow-up CT angiography revealed a T2EL in the proximal ascending aorta (Fig. 5A). The aneurysmal diameter further increased to approximately 60 mm (2 mm enlargement over 6 months) (Fig. 5B). The feeder vessel extended along the left vertebral body, with connections to the bronchial, costocervical, and left subclavian arteries (Fig. 5C). Chronic wall calcification related to Takayasu arteritis was present with no significant changes noted over time. Endovascular embolization was planned due to the progressive enlargement of the aneurysm and corresponding CT findings.

**Fig. 6:**
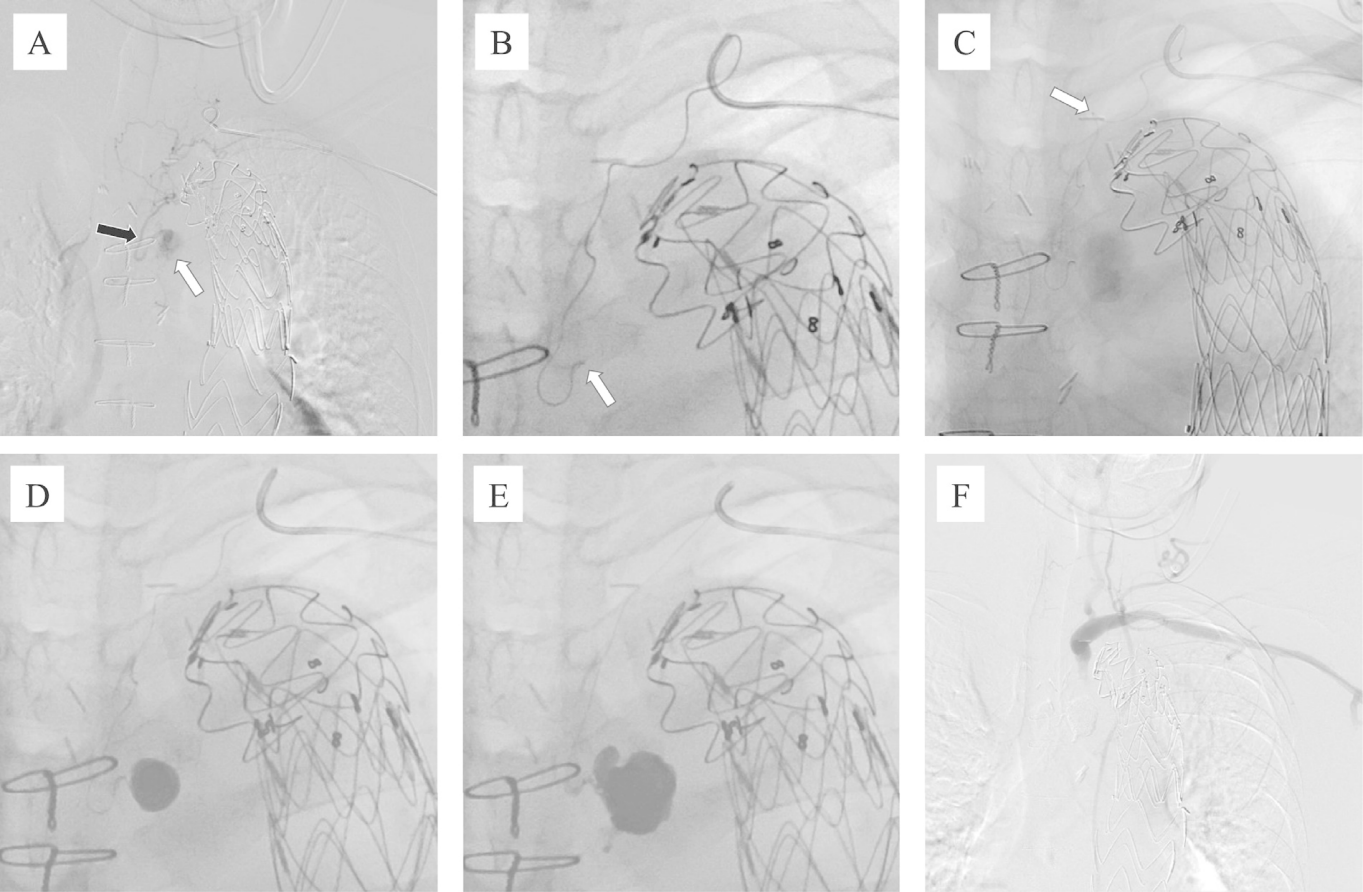
Embolization procedure in Case 3. A 5.3-Fr guiding sheath was introduced via the left brachial artery. (A) A 4-Fr RIM catheter is inserted into the left costocervical artery, and digital subtraction angiography (DSA) performed using a 2.85/2.9-Fr microcatheter demonstrated blood flow into the aneurysm sac (white arrow) through the bronchial artery (black arrow). (B) A 1.5/2.4-Fr (DeFrictor BULL, Medicos Hirata, Tokyo, Japan) microcatheter was advanced close to the aneurysm sac using a 0.010-inch (CHIKAI 10 and CHIKAI X010, Asahi Intecc, Aichi, Japan) guidewire, with the procedure performed at a right anterior oblique (RAO) working angle of 23°. (C) Severe vessel tortuosity resulted in narrowing of the microcatheter lumen (white arrow), complicating reintroduction of the microwire. (D, E) N-butyl cyanoacrylate (NBCA) was mixed with lipiodol (in a 1:2 ratio, approximately 3.6 mL) and injected to fill the sac and feeder vessels. (F) Post-procedure DSA confirmed complete exclusion of the endoleak, indicating successful embolization.

Under ultrasound guidance, the left brachial artery was accessed, and a 5.3-Fr guiding sheath was introduced. A 4-Fr RIM catheter was inserted using a 0.035-inch guidewire to access the left costocervical artery. Using a 0.014-inch (Meister, Asahi Intecc, Aichi, Japan) guidewire, a triple-coaxial system comprising a 2.85/2.9-Fr (Carry Leon High-flow, UTM, Aichi, Japan) microcatheter and 1.5/1.9Fr (Carry Leon Selective, UTM, Aichi, Japan) microcatheter was advanced. DSA revealed blood flow into the aneurysm sac via the bronchial artery (Fig. 6A). The RIM catheter was advanced proximally to support further microcatheter advancement; however, due to difficulty in advancing the microcatheter, it was exchanged for a 1.5/2.4-Fr (DeFrictor BULL, Medicos Hirata, Tokyo, Japan) microcatheter navigated with a 0.010-inch (CHIKAI 10 and CHIKAI X010, Asahi Intecc, Aichi, Japan) guidewire, which successfully reached the aneurysm sac (Fig. 6B). The sac measured approximately 27 mm in diameter. Severe vessel tortuosity led to a narrowing of the microcatheter lumen, making microwire reinsertion difficult (Fig. 6C). Coil embolization was considered unfeasible; therefore, NBCA mixed with lipiodol (33%) was injected, filling the sac and its feeder vessels (Fig. 6D and E). DSA confirmed successful embolization (Fig. 6F). Additional angiography of the right subclavian artery revealed no evidence of T2EL originating from its branches.

Contrast-enhanced CT performed 4 months after embolization revealed resolution of the treated T2EL; however, a new endoleak was identified. The feeding tract was unclear, making classification challenging. As there was no aneurysmal enlargement, conservative follow-up was adopted.

No major embolization-related complications were observed. In case 1, the procedure time, radiation dose, fluoroscopy time, contrast medium volume, and NBCA volume were 257 minutes, 596 mGy, 83 minutes, 66 mL, and 1.5 mL, respectively. In case 2, these values were 219 minutes, 1368 mGy, 54 minutes, 55 mL, and 1.8 mL (1.2 mL delivered from the sac to the left intercostal artery and 0.6 mL to the right intercostal artery). In case 3, they were 154 minutes, 624 mGy, 43 minutes, 64 mL, and 3.6 mL, respectively.

## Discussion

In this report, we successfully performed embolization using a flow-directed microcatheter and a 0.010-inch microwire in 3 cases of T2EL following TEVAR, achieving excellent immediate outcomes with NBCA alone or in combination with coils.

### Importance of sac embolization and microcatheter selection in endovascular treatment

In the endovascular treatment of T2EL following TEVAR, successful advancement of the microcatheter into the aneurysm sac is critically important. As demonstrated in our cases, the use of a flow-directed microcatheter (FDM) enabled the selective catheterization of small-caliber, highly tortuous vessels arising from the subclavian and bronchial arteries, an approach that had proven challenging with conventional catheters. A major advantage of the FDM is its flexibility and extremely small diameter, enabling easy navigation to distal vascular beds by utilizing blood flow, particularly within small and tortuous vessels [10]. Notably, in the embolization of cerebral arteriovenous malformations (AVMs), FDMs have facilitated super-selective embolization of extremely distal feeders that are inaccessible by conventional catheters [10].

However, the FDM was originally designed primarily for delivering liquid embolic agents such as NBCA and Onyx, and it has structural limitations that restrict coil delivery. In Case 3, severe vessel tortuosity and a sharp vascular curve resulted in a narrowing of the catheter lumen, limiting advancement of the coil pusher. Nonetheless, NBCA injection remained feasible and allowed for successful sac embolization. Proximal embolization of branches originating from the subclavian, intercostal, or bronchial arteries can potentially increase the risk of spinal ischemia due to the formation of collaterals with the spinal arteries. Although comprehensive data on the incidence of spinal cord infarction frequency after T2EL embolization following TEVAR are lacking, spinal cord ischemia has been reported in approximately 9% of cases involving segmental artery occlusion during TEVAR [11,12], and in up to approximately 5% of cases undergoing bronchial artery embolization for hemoptysis [13,14].

### Selection of embolic material: NBCA alone vs NBCA with coils

The selection of appropriate embolic materials influences long-term embolization efficacy. Recent studies indicate that the combination of coils and NBCA may offer superior mid- to long-term embolic efficacy compared to NBCA alone for T2EL following endovascular aortic repair (EVAR) [15]. Although these findings are based on studies of abdominal aortic aneurysm, they offer valuable insights into the long-term stability of liquid embolic agents, which may apply to T2EL following TEVAR.

NBCA is a liquid embolic agent that can be easily delivered through extremely fine microcatheters and is effective for filling aneurysm sacs inaccessible to coils, as illustrated in case 3. However, controlling NBCA injections can be challenging, with risks, including catheter adhesion and inadvertent embolization [16,17].

In contrast, combined embolization using coils and NBCA offers enhanced control, as the coils provide a framework that limits NBCA dispersion and confines the embolic material within the sac and feeder vessels [18,19]. In Case 2, the coils initially placed in the sac likely contributed to blood flow stagnation, facilitating effective sac filling with NBCA. Nevertheless, coil delivery can be challenging in narrow, tortuous vessels, increasing both procedural complexity and device costs. As demonstrated in Case 1, adequate embolization can be achieved with NBCA alone in a patient with a small sac volume (approximately 18 mm in diameter on DSA).

Regardless of embolization strategy, recurrence remains a critical issue. Although precise recurrence data for T2EL embolization following TEVAR are currently unavailable, studies on abdominal aneurysm following EVAR indicate recurrence rates of approximately 25% after direct sac puncture and over 60% following endovascular embolization, typically occurring within 2 years post-treatment [20,21].Furthermore, approximately 11% of these recurrent cases required additional intervention [8].These findings indicate a similarly significant risk of recurrence after TEVAR. In our series, case 2 developed a newly suspected T2EL originating from a different intercostal artery on follow-up imaging, and case 3 demonstrated a new endoleak 4 months after embolization, though its origin remained unclear. Despite the absence of aneurysm enlargement in both cases, these findings emphasize the critical importance of long-term surveillance, even after successful embolization. Due to our limited number of cases and relatively short follow-up duration, future multicenter studies with extended surveillance are essential to clarify recurrence risks and establish appropriate follow-up protocols for T2EL after TEVAR.

## Conclusion

We demonstrated that flow-directed microcatheters combined with 0.010-inch microwires facilitate catheter advancement into extremely tortuous, small-caliber feeder vessels and aneurysm sacs that are otherwise difficult to access following TEVAR. In all 3 cases, embolization using NBCA, either alone or in combination with coils, successfully eliminated T2EL in the short term. However, further studies with larger cohorts and longer follow-up periods are necessary to establish clear treatment guidelines and effective strategies for reducing the risk of recurrence and spinal ischemia.

## Patient consent

Written informed consent for the publication of this case report was obtained from the patients.
